# Therapeutic impact of vildagliptin vs. gliclazide on insulin resistance and advanced glycated end product levels in newly diagnosed Egyptian diabetics: a randomized controlled trial

**DOI:** 10.1007/s00228-025-03894-8

**Published:** 2025-08-05

**Authors:** Toka Elemary, Mohamed E. A. Abdelrahim, Mina Nicola, Dalia Zaafar

**Affiliations:** 1 Clinical Pharmacy Department, Faculty of Pharmacy, Modern University for Technology and Information, Cairo, Egypt; 2 Clinical Pharmacy Department, Faculty of Pharmacy, Beni-Suef University, Beni-Suef, Egypt; 3 Pharmacology and Toxicology Department, Faculty of Pharmacy, Modern University for Technology and Information, Cairo, Egypt

**Keywords:** Advanced glycated end products, Insulin resistance, Vildagliptin, Diabetes, Metformin, Clinical trial

## Abstract

**Background:**

The present study aimed to investigate the effects of vildagliptin, a dipeptidyl peptidase 4 inhibitor, on insulin resistance and weight reduction through advanced glycation end-product modulation in patients newly diagnosed with type 2 diabetes mellitus.

**Methods:**

This study was designed as a 12-week, randomized, controlled, parallel trial. A total of 120 patients with type 2 diabetes were selected and divided into two distinct groups: group I, patients who received gliclazide in combination with metformin, and group II, patients who received vildagliptin in combination with metformin. The percentage change in body weight was estimated along with serum advanced glycated end-product levels, glycated hemoglobin (HbA1c), and insulin resistance.

**Results:**

Upon completing the study period, when vildagliptin was added to metformin instead of gliclazide, the results demonstrated a significant improvement in insulin resistance, a downregulation of serum levels of advanced glycation end products, glycated hemoglobin, and a decrease in body weight.

**Conclusion:**

Vildagliptin showed a promising effect on improving type 2 diabetes mellitus–related complications by reducing advanced glycated end-product levels and insulin resistance. Additionally, vildagliptin reveals a favorable impact on weight reduction and glycated hemoglobin values.

**Graphical Abstract:**

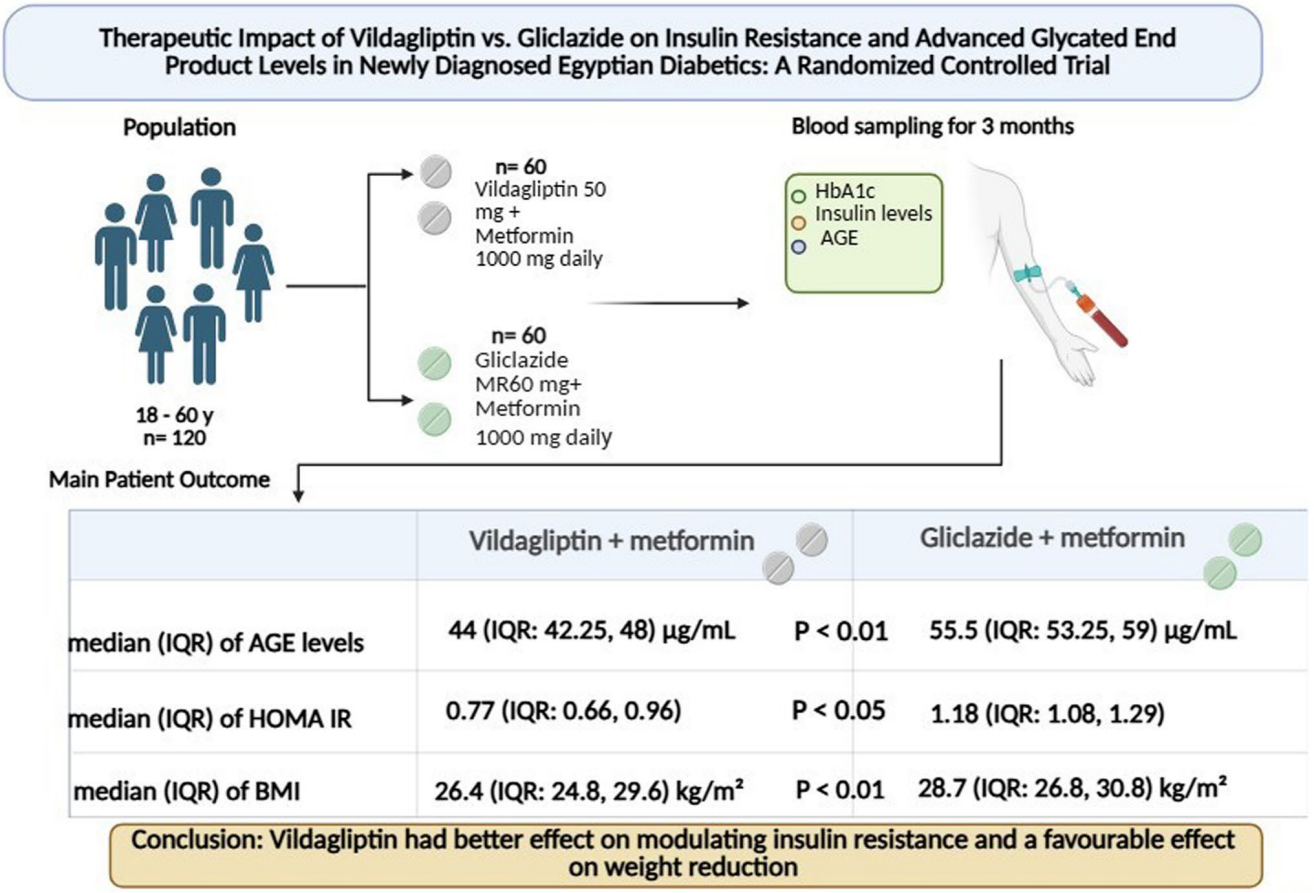

**Supplementary Information:**

The online version contains supplementary material available at 10.1007/s00228-025-03894-8.

## Introduction

Diabetes mellitus (DM) is a persistent metabolic condition characterized by elevated blood glucose levels and insulin resistance, presenting a formidable global health challenge in the twenty-first century [1]. The International Diabetes Federation reports a staggering 537 million individuals diagnosed with diabetes in 2021, projected to surge to 643 million by 2030 and 783 million by 2045 [2]. Notably, 90% of occurrences of diabetes are type 2 diabetes mellitus (T2DM) [3]. In Egypt, an estimated 10.9 million people had diabetes, which is anticipated to climb to 13 or even 20 million around 2030 to 2045. Egypt currently ranks eighth among countries with the most significant prevalence of diabetes and is anticipated to move up to sixth by 2045. Since 2003, the World Health Organization (WHO) and the International Diabetes Federation (IDF) have prioritized screening for the early detection of T2DM. Reasons for this decision include the high number of people who do not know they have diabetes (undiagnosed DM), the increasing global prevalence of diabetes and prediabetes, the fact that many patients experience complications especially microvascular ones before their diagnosis, and the fact that strict adherence to blood glucose monitoring is an effective way to slow the progression of diabetes-related health problems [4–6].

To improve control and reduce complications, researchers are investigating the underlying pathophysiology of T2DM, as well as studying various molecular pathways for antidiabetic drugs to prevent early onset, illuminating the pathophysiology underlying the development of T2DM’s complications, in which the long-term ramifications of uncontrolled hyperglycemia and insulin resistance play a pivotal role in precipitating macrovascular and microvascular complications, including diabetic nephropathy, retinopathy, cardiovascular risks, and cognitive impairment [1, 7–10]. Despite this, the precise pathogenesis of these complications remains incompletely definable. Emerging evidence suggests a correlation between stringent glycemic control and reduced risks of T2DM complications [11].

**Fig. 1:**
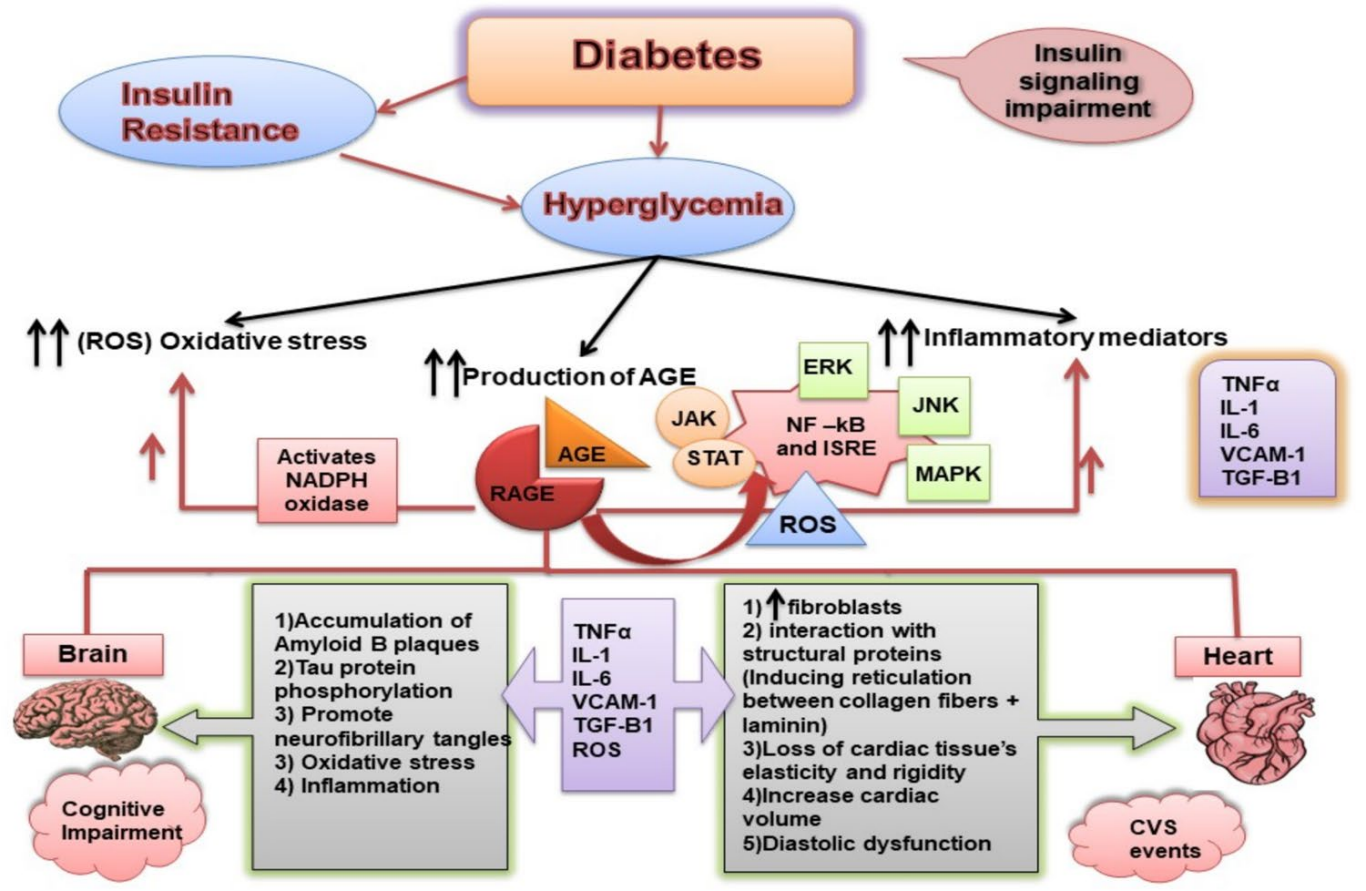
Signaling pathways in type 2 diabetes mellitus brought on by persistent hyperglycemia and insulin resistance. Whereas insulin resistance and hyperglycemia trigger 1, AGEs (advanced glycated end products); 2, oxidative stress (release of reactive oxygen species, ROS); and 3, inflammatory mediators (production of TNFα, interleukin-1, and 6), adherence, and growth factors (VCAM-1, vascular cell adhesion molecule-1, and TGB-1, transforming growth factor B), then after AGEs activate its receptors (RAGE), many pathways will be activated for inducing inflammation and also inducing the oxidative stress from these pathways activation of NF-κβ (nuclear factor kappa activator beta cells) and ISRE (interferon sensitive receptor element), which will activate pro-inflammatory cytokines TNFα (tumor necrosis factor-alpha), IL-1, and 6 (interleukin-1 and 6) and also induce the oxidative stress. NF-κβ and ISRE are activated by three pathways: the c Jun N-terminal kinase (JNK), extracellular signal-regulated kinase (ERK), and mitogen-activated protein kinase (MAPK) comprise the kinase pathway, the signal transducer and activators of transcription (STAT) and Janus kinase (JAK) pathway and ROS. Furthermore, because of RAGE stimulation, nicotinamide adenine dinucleotide phosphate (NADPH) oxidase activity produces ROS directly and indirectly. Inflammatory mediators and oxidative stress cause cell dysfunction and damage the cardiovascular system and brain, impairing cognition and causing CVS (cardiovascular s) events

Previous studies highlighted that chronic hyperglycemia and insulin resistance co-occurred to generate excessive reactive oxygen species (ROS), inducing high amounts of oxidative stress, advanced glycated end products (AGEs), and inflammatory mediators, as shown in Fig. 1. AGEs are reactive metabolites of persistent hyperglycemia associated with oxidative stress [11–17]. AGEs, as a variety of substances formed by oxidative processes, are involved in the development of DM-related problems. It has been found that during the chronic hyperglycemic conditions in DM, there are AGE receptors excessively expressed, along with the amount of AGEs formed in cells as a consequence of non-enzymatic condensation through oxidative stress and activation of several inflammatory pathways including protein kinase C pathway is higher than it is under normal conditions [18]. In Fig. 1, it is demonstrated that when AGEs bind to their receptor, the receptor of advanced glycated end-products (RAGE), they promote cellular development of ROS by stimulating NADPH oxidases, resulting in low-grade inflammation, insulin resistance, impaired endothelial function, stimulation of platelets, and the expression of the target gene for nuclear factor-κβ (NF-κβ) [18, 19]. Through these pathways and the multiple inflammatory cytokines that are stimulated, all these factors contribute to the occurrence of diabetic microvascular complications as well as macrovascular complications, which is consistent with prior studies that reported the association of elevated AGEs levels with diabetic-related microvascular complications, lipid profile, and obesity [20–23]. In addition, circulatory AGE concentrations were shown to be elevated in DM patients with CVD problems by 40 to 100% higher [24]. Thus, these circulatory biomarkers may predict a subsequent risk of diabetes [19].

Interestingly, AGEs have been reported to alter the protein structure of various macromolecules, affecting their functionality and initiating intracellular mechanisms that lead to the onset of inflammation, oxidative stress generation, mitochondrial dysfunction, endothelial damage, and thrombogenic outcomes in numerous types of cells through interaction with their RAGE receptors. These events can potentially promote certain systemic pathological conditions, including hypertension, retinopathy, nephropathy, cardiovascular complications, and cognitive dysfunction [10, 14, 19, 25–31], as illustrated in Fig. 1. Therefore, AGEs are considered key biomarkers that contribute to the development of different diabetic complications.

Obesity is a potential factor that can contribute to the development of insulin resistance. However, the precise mechanism is uncertain; obesity is a key factor in the prolonged, low-grade systemic inflammation that leads to high insulin levels and insulin resistance [32]. Obesity can induce multiple inflammatory mediators, such as tumor necrosis factor-alpha (TNF-α), which is crucial for the development of insulin resistance and T2D [32–35]. Moreover, obesity plays a significant role in the development of T2DM comorbidities [35, 36].

Nowadays, there has been a growing interest in repurposing antidiabetic medications not only for managing glucose homeostasis but also for addressing diabetes-related complications [37–39] to achieve better control. Dipeptidyl peptidase-4 inhibitors (DPP4Is) have become a promising new treatment for T2DM due to their significant ability to regulate glycated hemoglobin [40, 41]. One of this class most promising members, vildagliptin, can be employed as a single therapy or in addition to other oral hypoglycaemic agents. Its favorable efficacy profile, coupled with a low incidence of hypoglycemia, absence of weight gain, and minimal cardiovascular event risk, underscores its therapeutic significance as an antidiabetic drug for type 2 DM treatment [42, 43].

The primary objective of this randomized clinical trial is to explore the therapeutic effect of combining vildagliptin and metformin on glycemic management and insulin resistance in recently diagnosed Egyptian individuals with T2DM, and to determine whether early detection and care could prevent T2DM-related problems from developing in Egyptian diabetic patients. Specifically, we concentrated on the changes in serum AGEs, which are a crucial biochemical indicator of inflammation and oxidative stress associated with diabetes. Since AGEs play a vital role in the development and progression of insulin resistance, as well as diabetes complications, understanding their regulation by vildagliptin provides valuable insight into its potential positive effects beyond glycemic control.

### Study outcomes

The primary aim was to enhance insulin sensitivity and reduce the serum levels of AGEs in individuals with recently diagnosed T2DM by implementing appropriate antidiabetic therapy. Secondary objectives included evaluating the influence of the medication on glycemic management and body weight.

## Material and methods

### Drugs

Metformin was sourced as commercially available Cidphage® tablets (1000 mg) from CID, Egypt. Gliclazide was sourced as commercially available Diamicron® MR tablets (60 mg) by Servier, France. Vildagliptin was acquired as a commercially available Icandra® tablets (50 mg) from Mesh Premiere, Egypt.

### Study design and participants

The present study was a 3-month randomized, double-blind, controlled trial, carried out at the National Institute of Diabetes and Endocrinology. Participants included individuals diagnosed with T2DM. The research study comprised multiple phases: a 2-week screening visit followed by a 3-week wash-out period during which metformin was the only medication used, adhering to established protocols. Subsequently, a 3-month treatment period follows.

### Ethics approval

The study received ethical approval from the Research Ethics Committee of the National Institute of Diabetes and Endocrinology (approval no. IDE00281) in April 2022. Before participation, written informed consent was obtained from all individuals following ethical guidelines outlined in the Declaration of Helsinki [44].

The trial adhered to strict ethical principles throughout its course and was registered at clinicaltrial.gov under the identifier NCT05429554 on June 19, 2022. Inclusion criteria: individuals between the ages of 18 and 77 years; diagnosed with T2DM within the past 2 years. T2DM was diagnosed using plasma glucose parameters and HbA1C criteria [45, 46], with a verified HbA1c level of ≥ 6.5. Diabetic patients with a BMI of 22 to 40 kg/m^2^ were also included. Before enrollment, participants received guidance on lifestyle modifications, including dietary counseling and exercise training.

Exclusion criteria: individuals who had used weight-loss medications within the previous 3 months; individuals with persistent congestive heart failure (NYHA Classification III − IV), chronic liver disease, cerebrospinal diseases, dementia, Alzheimer’s disease, pregnant, or nursing individuals; or those with hypersensitivity to metformin or DPP-4Is.

### Randomization and masking

Participants were randomized in a 1:1 ratio to either the vildagliptin + metformin or gliclazide + metformin groups using simple randomization with the help of computer-validated software (Random allocator). Treatment allocation was concealed from patients, researchers, clinicians conducting the evaluations, and data analysts. The allocation of treatment was kept hidden during the data processing phase, maintaining the study’s integrity. According to the intention-to-treat principle, all randomized individuals were included in the analysis based on their initial allocation to groups, regardless of adherence or withdrawals.

### Patient classification and pharmacological treatment plan

The study started in November 2022 and ended in November 2023. Initially, 150 participants were recruited for the study; however, 30 individuals were excluded because they did not meet the inclusion criteria. The remaining 120 participants were enrolled and randomized into two groups, each with 60 participants:

- Group I (*n* = 60): gliclazide + metformin group, Participants received gliclazide MR 60 mg/day before breakfast along with metformin 1000 mg/day with lunch. As previously shown in earlier studies, gliclazide MR 60 mg/day has proven efficacy in glycemic control [47, 48].

- Group II (*n* = 60): vildagliptin + metformin group. Participants administered vildagliptin 50 mg/day before breakfast along with metformin 1000 mg/day with lunch [49, 50]. In multiple earlier studies, a 50 mg dose of vildagliptin administered once daily has shown a similar therapeutic benefit to the twice-daily dose, especially when combined with metformin [52–55].

**Fig. 2:**
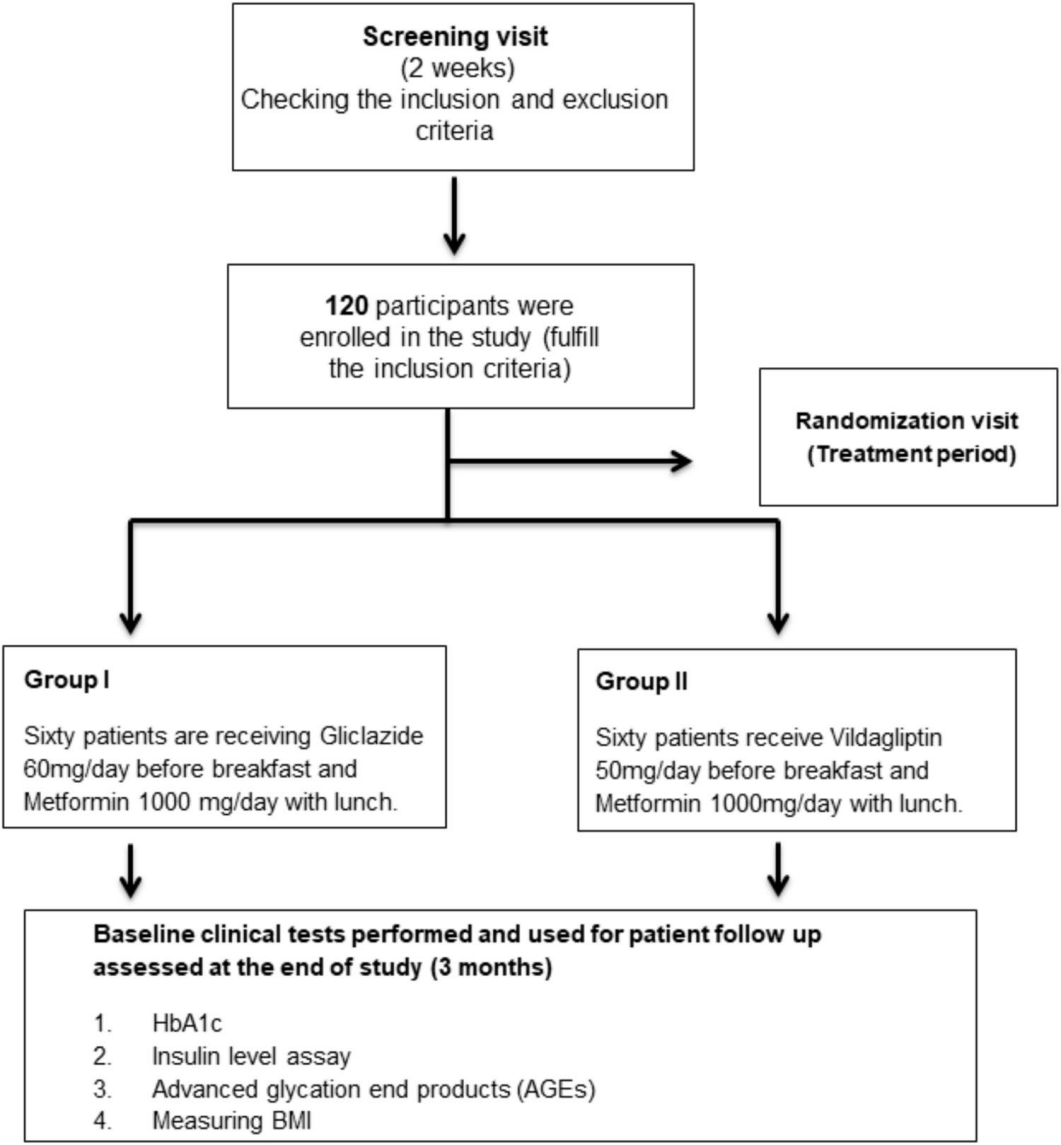
A flowchart illustrates the design of the study, the grouping of participants, and the parameters evaluated throughout the research. The schematic outlines the structure of the study, detailing how participants were categorized and the various factors measured during the investigation. HbA1c, glycated hemoglobin; AGEs, advanced glycated end products; BMI, body mass index

Both groups completed the study without any dropouts. The prescribed treatment regimen was followed through to the trial completion (Fig. 2). Blood samples were taken from each participant at the study initiation and upon completion.

### Parameters assessed

Throughout the study, participants underwent weekly telephone monitoring to evaluate any discomfort. Various parameters were assessed, including the following:

1. Serum insulin and glucose levels: Regularly measured while fasting [51, 56] at the initiation and the end of the study to monitor metabolic parameters.
2. HbA1C: Obtained at the initiation and end of the study for a comprehensive assessment of long-term glucose control.
3. BMI was estimated at the start and the end of the study using the formula: $BMI=Weight(kg)/Height^2\;(m^2)$  
4. The percentage change in body weight (% change in BWT) was estimated at the end of the study using the formula: % change in BWT = [(Final BWT − Basal BWT)/Basal BWT] × 100 [57].
5. Serum levels of AGEs: Evaluated at the start and the end of the study for a comprehensive insight into glycation-related processes.

### Blood sample processing

Within 30 min of collection, blood samples were centrifuged at 2000 × g for 15 min. The sera were separated and stored at – 20 °C until needed for various tests [58].

### Assay kits for enzyme-linked immunosorbent

The serum levels of AGEs, insulin, and HbA1C were estimated through the enzyme-linked immunosorbent technique (ELISA) using the following kits: #SL0077Hu purchased from Sunlong Biotech Co. (Hangzhou, China), (#01.09.4A.01.01.02) purchased from Zybio Co. (Chongqing, China) and #401,003 obtained from the Egyptian Company for Biotechnology (Cairo, Egypt), respectively, following the manufacturers’ instructions.

#### Determination of serum advanced glycated end products (AGEs)

Serum AGEs were assessed by an enzyme-linked immunosorbent assay (ELISA) following the manufacturer’s directions. A standard curve was created utilizing AGE concentrations ranging from 26 to 1600 ng/mL. The assay’s detection limit was 5 ng/mL, and the intra-assay coefficient of variation (CV) was less than 10%, guaranteeing measurement accuracy between duplicates. The inter-assay CV was less than 12%, indicating consistency among several runs.

### Insulin resistance

Insulin resistance was evaluated using the homeostatic model assessment for insulin resistance (HOMA-IR) index, estimated by the following formula: HOMA-IR index = [fasting glucose (mg/dL) × fasting insulin (mU/mL)]/405, as described earlier by Matthews et al. [59].

### Personalized lifestyle adjustment

Each participant received detailed guidance on lifestyle modifications, including planned recommendations for physical exercise and personalized dietary recommendations regulating carbohydrate and fat intake [60].

### Subject adherence and analysis of safety

Patient compliance with the study medication and any potential adverse events were assessed through weekly phone conversations and monthly interviews, which included focused questions for patients. During monthly scheduled visits, compliance was assessed by recording the number of pills taken [41].

### Statistical analysis

The ANZMTG Power Calculator online tool was used for calculating the sample size. Considering a change in AGE levels of 0.85 µg/mL between treatment groups as clinically relevant, with a standard deviation of 0.25 µg/mL. Using a two-sided independent samples *t*-test, with a type I error of 0.05 and a power of 80%, the required sample size was calculated to be 96 participants. We inflated the sample size to account for potential dropouts, resulting in a total sample size of 120 patients (60 per group).

The intention-to-treat (ITT) analytic set was used to examine the primary and secondary outcome variables. All randomized patients who received the study drug dose and had a non-missing baseline assessment for the effectiveness variable were included. All findings were systematically tabulated and subjected to a normality check by the Shapiro–Wilk test. Non-normally distributed data were represented as median and interquartile range (IQR). Group comparisons utilized the Mann–Whitney *U* test and the Wilcoxon signed-rank test. The variance between groups at baseline or study endpoint was assessed through the Mann–Whitney U test. On the other hand, changes within each group from baseline to the 12-week follow-up were inspected via the Wilcoxon signed-rank test for continuous parameters. Data analysis was performed using GraphPad Prism software, version 9, with a statistical significance level of *P* < 0.05.

Multivariate regression analysis was conducted to determine correlations between changes in AGE levels as the dependent variable and the following variables: age, BMI, baseline HbA1c, treatment group (group I vs. group II), and gender. The variance inflation factor (VIF) and tolerance statistics were computed to evaluate multicollinearity among independent variables. SPSS software (SPSS, Chicago, IL) was used to perform regression.

## Results

### Baseline demographics and clinical features

Table 1 provides an overview of baseline clinical and demographic data for the participants. In group I (metformin + gliclazide), 55% of participants identified as male, whereas in group II (vildagliptin + metformin), the proportion of male participants was 56.7%. Noteworthy, there were no statistically significant variances between the two cohorts concerning age, BMI, HbA1c levels, insulin concentrations, and AGE levels at baseline. The median age within group I was 53 years (46–58), while in group II, it was 54 years (48–58). BMI analysis revealed that most participants in both groups fell within the overweight category (25–29.9 kg/m^2^).

**Table 1:** The baseline and demographic features of the study population

Characteristics	Group I Metformin + Gliclazide	Group II Vildagliptin + Metformin	*P* value
Sex			
Female Male	27 (45%) 33 (55%)	26 (43.3%) 34 (56.7%)	––
Age (years)			
Median (IQR) < 51 51–59 > 59	53 (46—58) 26 (43.3%) 24 (40%) 10 (16.7%)	54 (48—58) 24 (40%) 25 (41.7%) 11 (18.3%)	0.5218
HbA1c (%)			
N Median (IQR) < 7% ≥ 7%	60 7.5 (7.2—8.1) 5 (8.3%) 55 (91.7%)	60 7.65 (7.4—8) 6 (10%) 54 (90%)	0.6380
HOMA-IR			
Median (IQR)	1.18 (1.04–1.37)	0.89 (0.74–1.05)*	< 0.0001
BMI (Kg/m^2^)			
Median (IQR) < 25 25–29.9 ≥ 30	27.95 (26—30.3) 13 (21.7%) 32 (53.3%) 15 (25%)	27.65 (25.75—30.3) 10 (16.7%) 35 (58.3%) 15 (25%)	0.2518
Fasting insulin level (mIU/ml)			
Median (IQR)	2.85 (2.63—3.1)	2.95 (2.53—3.28)	0.3946
Serum AGEs levels (µg/mL)			
Median (IQR)	54 (50—59)	54.5 (51—57.8)	0.7147
Weight (Kg)			
Median (IQR)	80.75 (74.2—90)	80 (71.3—89)	0.3175
Smoker Non- Smoker	12 (40%) 18 (60%)	14 (46.7%) 16 (53.3%)	–––
Hypertensive Non- Hypertensive	19 (63.3%) 11 (36.7%)	13 (43.3%) 17 (56.7%)	––-

Group I: Gliclazide 60 mg/day + metformin 1000 mg/day; group II: vildagliptin 50 mg/day + metformin 1000 mg/day. Results were presented as *n* (%) and median and interquartile range (IQR) and assessed using Mann–Whitney U test

*Significantly distinct from group I at *p* < 0.05

*BMI*, body mass index; *HbA1c*, glycated hemoglobin; *AGEs*, advanced glycated end products; *HOMA-IR*, homeostasis model assessment-estimated insulin resistance

### Estimation of changes in insulin levels after adding gliclazide or vildagliptin to metformin treatment

**Fig. 3:**
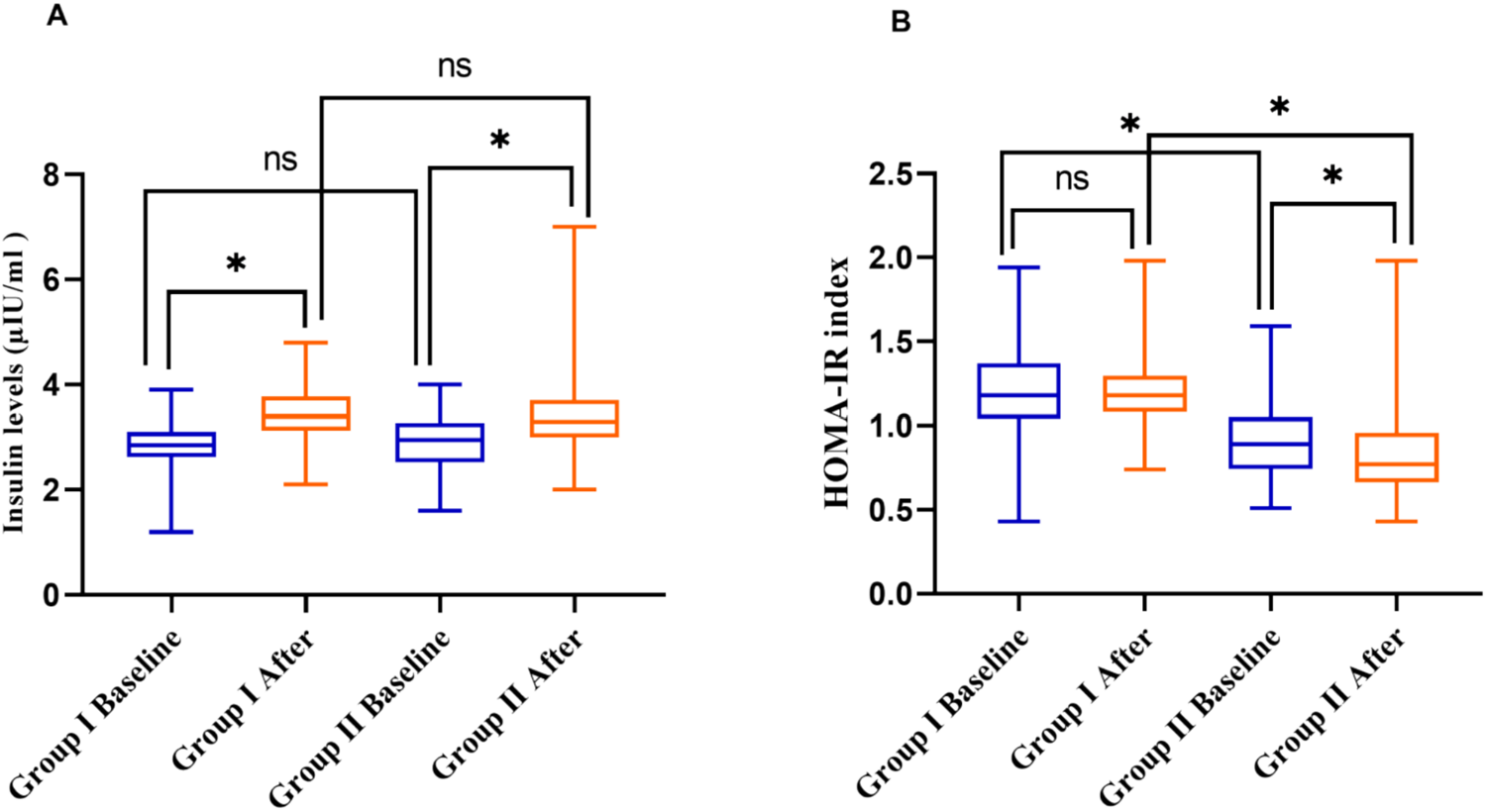
The effects of adding vildagliptin or gliclazide to metformin on glycemic profile. **A** Insulin levels and **B** HOMA-IR index, in diabetic patients after 12 weeks of treatment. Group I, patients in group I received gliclazide 60 mg daily and metformin 1000 mg daily. Group II, patients in group II received vildagliptin 50 mg daily and metformin 1000 mg daily. The Wilcoxon and Mann–Whitney *U* test was performed. Data were represented as median values with interquartile ranges. HOMA-IR, homeostasis model assessment-estimated insulin resistance, *Differs significantly from group I; ns is non-significantly at *p* < 0.05

Table S1 presents the changes in the measured parameters that occurred during the 12-week trial period. During screening, it was observed that insulin levels in group I elevated from 2.85 (2.62–3.1) to 3.4 mIU/mL (3.12–3.77), and in group II, from 2.95 (2.52–3.27) to 3.3 mIU/mL (3–3.7) after a 12-week treatment period. Despite the noted elevation in insulin levels, there was no statistically significant difference between the two groups (Table S1, Fig. 3A).

### Estimation of changes in HbA1c and HOMA-IR after adding gliclazide or vildagliptin to metformin treatment

As shown in Table S1, the current findings demonstrated a considerable alteration in HbA1c levels within both intervention groups over the 12-week treatment duration. At baseline, HbA1c levels were 7.5% (7.2–8.1) in group I and 7.65% (7.4–8) in group II. By the end of the 12 weeks, HbA1c levels had declined to 6.7% (6.22–7.07) in group I and 6.3% (6.1–6.6) in group II. Importantly, patients in group II experienced a significant (*P* < 0.05) decrease in HbA1c from baseline to endpoint compared to those in group I. Notably, 82% of patients in group II achieved HbA1c levels below 7%. In contrast, in group I, this percentage was 73% (Fig. 5A). Additionally, both treatment groups exhibited significant reductions in HbA1c levels following the 12-week treatment period compared to their respective baseline values.

Furthermore, insulin resistance was assessed in both groups using the HOMA-IR. Our findings highlighted a significant reduction in the estimated HOMA-IR after the 12-week treatment duration in patients of group II, from 0.89 (0.74–1.05) to 0.77 (0.66–0.96) (*P* < 0.05). Conversely, in group I, the combination of gliclazide and metformin did not exhibit any noticeable difference in lowering calculated HOMA-IR, with values ranging from 1.18 (1.04–1.37) to 1.18 (1.08–1.29), as shown in Fig. 3B and Table S1.

### Estimation of changes in body mass index and weight after adding gliclazide or vildagliptin to metformin treatment

**Fig. 4:**
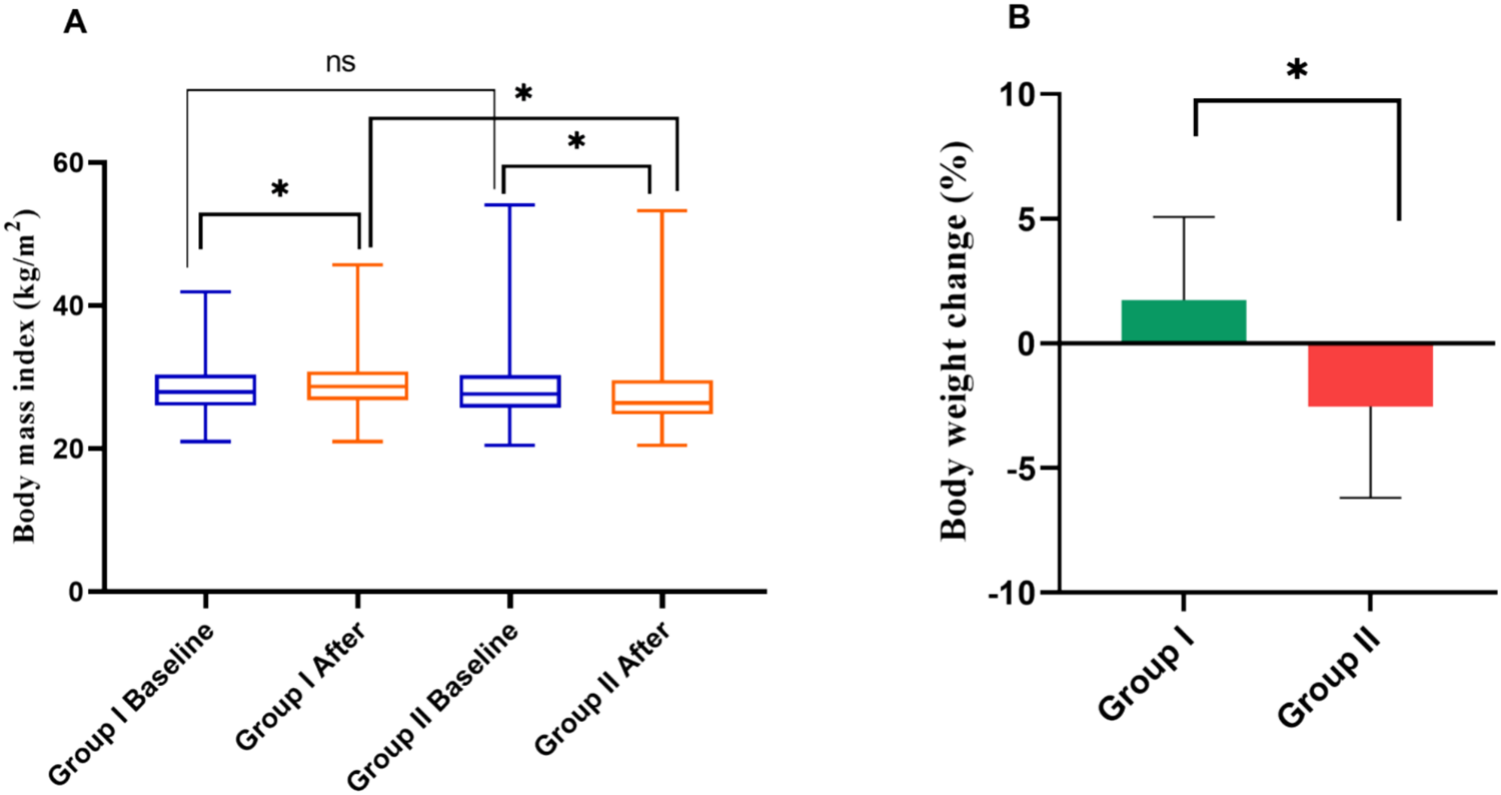
The effect of adding vildagliptin or gliclazide to metformin on **A** body mass index (BMI, kg/m.^2^) after 12 weeks of treatment and** B** percent change in body weight (%). Group I, patients in group I received gliclazide 60 mg daily and metformin 1000 mg daily. Group II, patients in group II received vildagliptin 50 mg daily and metformin 1000 mg daily. The Wilcoxon and Mann–Whitney *U* tests were performed. Data were represented as median values with interquartile ranges. Percentage change in body weight was estimated utilizing a formula: % change BWt = [(final BWt − baseline BWt)/baseline BWt] × 100. *Differs significantly from group I; ns is non-significantly at *p* < 0.05

Table S1 reveals that, by week 12, the BMI in group I exhibited an increase from 27.95 (26–30.35) to 28.7 kg/m^2^ (26.8–30.8), whereas in group II, it decreased from 27.65 (25.75–30.3) to 26.4 kg/m^2^ (24.8–29.6), which is represented in Fig. 4A. Notably, the reduction in group II was statistically significant, demonstrating a noteworthy difference between the two groups. In Table S2, the findings further reveal that the percentage change in body weight in group II (− 2.58% (− 4.53, 0)) was significantly higher than in group I (2.12% (0, 3.78)) (Fig. 4B, Table S2).

### Estimation of changes in serum levels of advanced glycated end products after adding gliclazide or vildagliptin to metformin treatment

**Fig. 5:**
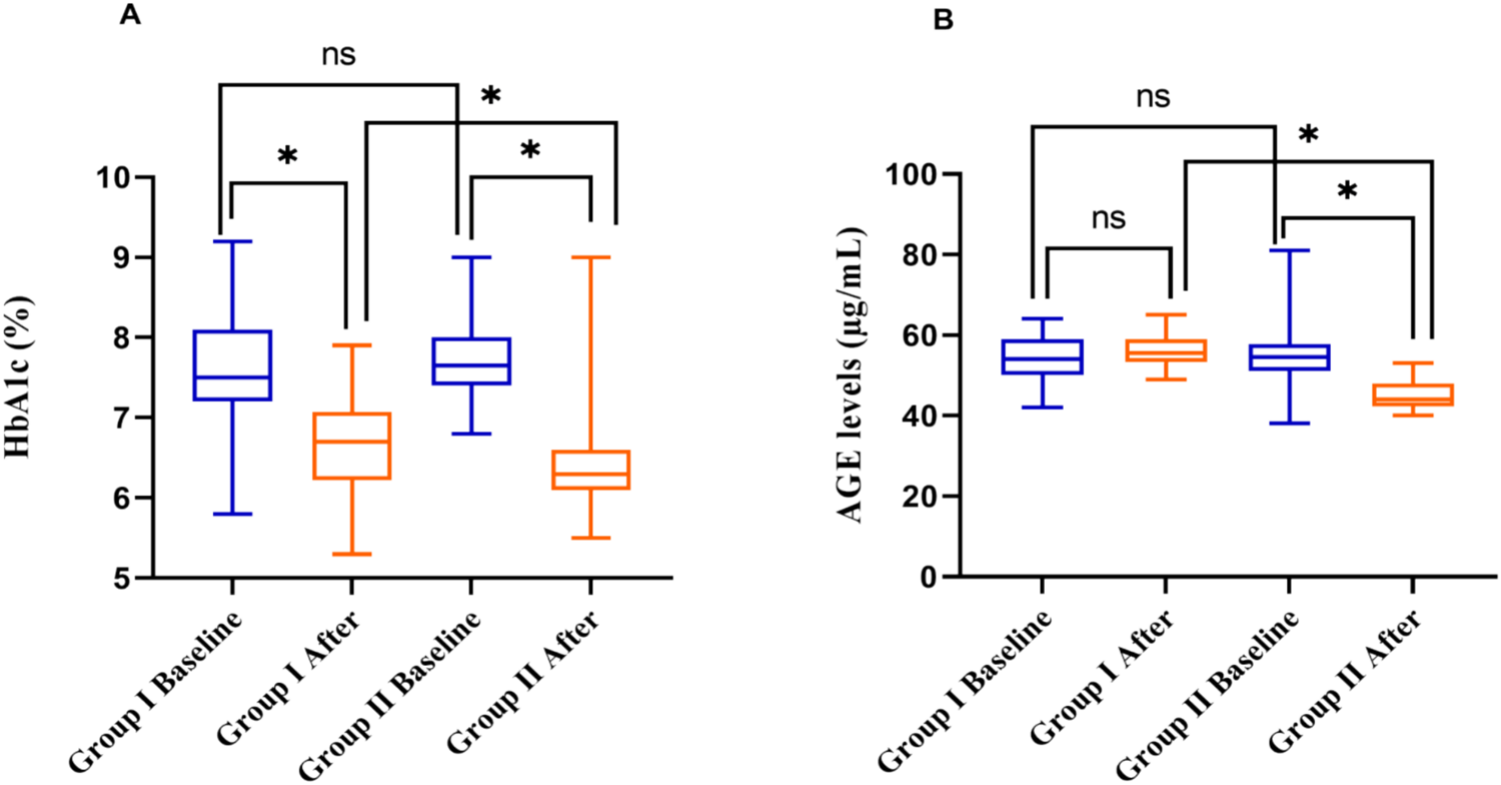
The effect of adding vildagliptin or gliclazide to metformin on **A** glycated hemoglobin (HbA1c %) and **B** serum levels of advanced glycated end products (AGEs, µg/mL) after 12 weeks of treatment. Group I, patients in group I received gliclazide 60 mg daily and metformin 1000 mg daily. Group II, patients in group II received vildagliptin 50 mg daily and metformin 1000 mg daily. The Wilcoxon and Mann–Whitney *U* tests were performed. Data were represented as median values with interquartile ranges. HbA1c, glycated hemoglobin; AGE, advanced glycated product. *Differs significantly from group I; ns is non-significantly at *p* < 0.05

Table S1 has demonstrated a significant difference in AGE serum levels among the two treatment groups after the treatment period. The addition of gliclazide to metformin therapy did not result in a reduction in AGE levels among patients, with values of 55.5 µg/mL (53.25–59) compared to baseline values of 54 µg/mL (50–59) µg/mL. Conversely, the addition of vildagliptin to metformin therapy led to a notable decrease in AGE levels, with values of 44 µg/mL (42.25–48) compared to baseline values of 54.5 µg/mL (51–57.75) (*P* < 0.05), as shown (Fig. 5B, Table S1). This decrease in AGEs is especially notable, as AGEs are suspected of contributing to insulin resistance by disrupting insulin signaling pathways. The noted reduction in AGE levels correlates with the improved clinical outcomes, such as lowered HbA1c and fasting glucose levels, underlining vildagliptin’s therapeutic promise in modifying both glycemic control and the cause of insulin resistance.

Table 2 underscores the results of the multivariable linear regression model conducted, which adjusted for covariates including age, BMI, gender, and baseline HbA1c. The current findings revealed that group I treatment was significantly associated with a greater reduction in AGE levels compared to group II treatment (*B* = 10.96, *p* < 0.001). None of the other covariates significantly influenced AGE outcomes. No multicollinearity was detected (all VIF < 1.2) (Table 2).

**Table 2:** Multivariable linear regression model for predicting changes in AGE serum levels following treatment

Variable	*B* coefficient	SE	B coefficient standardized	*P* value	95% CI		VIF
Treatment	10.955	1.219	0.638	** < 0.001**	8.541	13.369	1.007
Age	0.023	0.086	0.019	0.788	− 0.147	0.193	1.041
BMI	− 0.216	0.139	−0.115	0.121	− 0.490	0.058	1.092
Gender	0.876	1.293	0.051	0.499	− 1.685	3.436	1.117
Baseline HbA1C	0.161	1.062	0.011	0.880	− 1.943	2.266	1.014

Treatment are gliclazide 60 mg/day + metformin 1000 mg/day or vildagliptin 50 mg/day + metformin 1000 mg/day

*SE*, standard error; *95% CI*, 95% confidence interval; *VIF*, variance inflation factor; *BMI*, body mass index

### Evaluation of the safety and tolerability after adding gliclazide or vildagliptin to metformin treatment

Table 3 outlines the predominant adverse events documented over the 12-week duration. Thirteen AEs were reported, with five (8.3%) occurring in group I: hypoglycemia, nausea, dizziness, constipation, and peripheral edema. Group II reported four AEs (6.7%), including headache, decreased appetite, tremors, and fatigue. All AEs reported in both treatment groups were classified as mild, with no unexpected safety concerns identified. Both groups experienced a comparable incidence of AEs. Importantly, none of the patients discontinued treatment due to AEs. No significant SAEs or deaths were observed throughout the study. According to statistical analysis, the incidence of AEs did not significantly differ between the two groups.

**Table 3:** Predominant adverse events reported by patients in the two treatment groups

	**Group I**	**Group II**
AE	*n* (%)	
Headache	-	1 (1.67%)
Hypoglycemia	1 (1.67%)	-
Nausea	1 (1.67%)	-
Decrease appetite	-	1 (1.67%)
Tremor	-	1 (1.67%)
Dizziness	1 (1.67%)	-
Fatigue	-	1 (1.67%)
Nasopharyngitis	-	-
Constipation	1 (1.67%)	-
Upper respiratory tract infection	-	-
Arthralgia	-	-
Hyperhidrosis	-	-
Oedema peripheral	1 (1.67%)	-
Total AEs	5	4

Data are in *n* (%). Group I (gliclazide 60 mg daily tablets + metformin 1000 mg daily tablets); group II (vildagliptin 50 mg tablets + metformin 1000 mg daily tablets)

*AEs*, adverse events; *UTI*, urinary tract infection

## Discussion

The current study is a randomized controlled clinical trial designed to evaluate the therapeutic impact of vildagliptin on insulin resistance and serum levels of AGEs in Egyptian T2DM patients for 12 weeks through several assessments. The present findings revealed that, upon adding vildagliptin to metformin therapy for diabetic patients, there was a general enhancement in their glycemic control. Examining the glycemic profile data in more detail revealed that adding vildagliptin to metformin had a more robust efficacy in regulating insulin levels, lowering FPG and HbA1c than gliclazide with metformin. Also, group II showed that the percentage of patients who achieved HbA1c < 7% was higher than in group I. The reduced HbA1c and FPG can serve as evidence of this improvement, in addition to body weight loss, compared to the gliclazide and metformin-treated group.

The notable reduction in HOMA-IR in group II compared to group I indicated better modulation of insulin resistance. These promising findings were in line with several other randomized studies that reported the beneficial effect of vildagliptin in controlling body weight, FPG, and HbA1c [41, 56, 62–64]. Moreover, Mohan’s study, an observational, retrospective, real-world evaluation study, highlighted that the combination of vildagliptin with metformin resulted in better glycemic control than metformin single therapy, as the combination arm demonstrated a more significant reduction in HbA1c levels than the metformin-only arm [65]. Also, it was shown previously that the occurrence of hypoglycemic events is one of the adverse events of using gliclazide as a hypoglycemic therapy [66–68].

However, the present study observed a superior enhancement in insulin sensitivity in the group that received vildagliptin over the group that received gliclazide. This may be due to how DPP-4 inhibitors affect GLP-1; they prevent GLP-1 from being broken down, thereby increasing insulin synthesis and decreasing glucagon secretion in response to elevated plasma glucose levels. Additionally, DPP-4 inhibitors have been shown to exhibit significant reductions in HbA1c [69]. Conversely, by blocking KATP channels, gliclazide causes the pancreatic beta cells to release more insulin. This process is not glucose dependent. Regardless of blood glucose levels, it promotes insulin secretion, which raises the possibility of hypoglycemic episodes and their consequent adverse events, especially in elderly diabetic patients [70–72].

Obesity is considered one of the primary risk factors for type 2 diabetes mellitus (T2DM). In our study, adding vildagliptin to metformin in group II revealed a substantial decrease in BMI after 12 weeks of treatment compared to the gliclazide addition to metformin in group I. The present findings were in the same line as several previous reports, which outlined that either with or without metformin, vildagliptin caused a drop in body weight [50, 56, 73]. The current findings disagreed with some earlier research that suggested vildagliptin had a neutral effect on body weight reduction. These investigations reported a non-significant change in BMI in the vildagliptin-treated group [74, 75]. Additionally, our results showed that the gliclazide-treated group revealed an increase in body weight after 12 weeks, which was consistent with Stubbs and colleagues’ findings. However, Uddin and colleagues demonstrated that gliclazide has a neutral effect on body weight [71, 76]. The discrepancy between the results and the authors mentioned above may be explained by variations in the population, procedures utilized, racial differences, or study duration.

Furthermore, gliclazide was found to slightly elevate AGE levels, although it did not show a significant rise. It reflects the adverse effect of gliclazide on modulating serum AGE levels in people with diabetes. Importantly, vildagliptin succeeded in downregulating the serum levels of AGEs, which were reported to have a negative impact on diabetic patients as cardiovascular complications, neurodegenerative diseases, and diabetic retinopathy [77–79]. While aging, tiny amounts of AGEs, the result of Millard’s reaction, build up in skin, cartilage, and pericardial fluid [76]. Several studies have reported that elevated serum levels of AGEs and other markers of insulin resistance, such as FPG and HOMA index, are significant contributors to insulin resistance [13, 80, 81]. The damaging effects of AGEs are exacerbated in patients with T2DM as AGE production is increased by chronic hyperglycemia [82–84]. The increased level of AGEs increases binding to its receptor (RAGE) as well as the production of DPP-4 enzymes [85], which in turn causes inflammation by raising intracellular ROS production and stimulating NAPDH oxidases, insulin resistance, impaired endothelial function, and activation of platelets as demonstrated in Fig. 1 and mentioned previously in several reports [80, 86].

According to our study findings, vildagliptin dramatically lowered serum AGE levels, a substantial finding given that AGEs contribute to the development of diabetes-related problems. As previously discussed, AGEs worsen vascular damage and insulin resistance through promoting oxidative stress and inflammation. This is corroborated by earlier studies that found that AGEs worsen insulin production, interfere with insulin receptor substrate signaling, impair insulin resistance, and increase the production of oxidative stress, which in turn causes inflammation that may contribute in the development of several diabetes-related problems as retinopathy, nephropathy, and CVD [87–89]. Consequently, the decrease in AGEs observed in this study is a significant indication of reduced inflammatory and oxidative stress in diabetic individuals rather than merely a biochemical improvement. Thus, the improvement in the trial’s clinical results, such as lowered HbA1c and fasting glucose levels, and modulated insulin resistance, may be connected to the reduction in AGE levels. Our results align with a previous preclinical investigation that linked the decrease in AGE levels to the adjustment of insulin resistance [90]. Vildagliptin’s ability to lower AGE levels may protect against long-term diabetic complications, confirming its role as an essential treatment option in managing diabetes. This finding is consistent with broader research indicating that AGE-lowering therapies can enhance metabolic health by alleviating AGE-related tissue damage [37, 91–94].

Furthermore, metformin was found to indirectly influence AGEs by lowering early glycation products, including fructosamine and methylglyoxal, which function as glycation intermediates before producing AGEs and oxidative stress products, hence reducing AGEs [95]. Thus, the combination of vildagliptin plus metformin significantly impacted the reduction of AGE levels and modulation of insulin resistance. Apart from the outcomes of our investigation, it is noteworthy to mention the results of the VERIFY study, which showed that the extended use of vildagliptin in conjunction with metformin considerably decreased the progression of diabetes and the frequency of complications over 5 years, compared to metformin monotherapy [96].

This critical action of vildagliptin makes it a promising agent for reducing and controlling diabetic complications in addition to its glycemic-lowering effect. Furthermore, DPP-4 inhibitors are generally well tolerated and have no SAEs.

Although the 12-week trial was not designed to evaluate long-term effects, the reduction in AGEs that vildagliptin resulted in, as well as its weight reduction effect, may offer a mechanism by which the protective effects observed in longer-term studies could be explained. Further studies should investigate the role of AGEs in regulating long-term diabetes outcomes. Moreover, comparative studies evaluating the impact of combination therapies versus monotherapy with metformin would be valuable in enhancing treatment strategies.

Despite the promising outcomes observed, certain limitations should be mentioned. These involve the single-center study, short duration, small numbers of participants, and a lack of long-term follow-up, which are constraints that future studies should strive to overcome to build on these preliminary findings. Since no institution funded the study, we lacked access to several critical laboratory data, including lipid profiles, eGFR, and biomarkers of inflammation and oxidative stress. By incorporating these findings into future research, we may gain a better understanding of how medications affect the progression of complications in T2DM.

Long-term investigations are necessary to determine whether these effects lead to a persistent reduction in diabetic complications. Our data suggest that the reduction in AGEs with vildagliptin may help mitigate these risks, as AGEs are associated with the development of diabetes complications; however, further research is needed to confirm this hypothesis.

## Conclusion

The present study delved into the comparative efficacy of augmenting metformin therapy with either vildagliptin or gliclazide in managing hyperglycemia among newly diagnosed patients. The findings suggest that vildagliptin may offer advantages over gliclazide in glycemic control, potentially due to its effects on fasting plasma glucose levels, insulin sensitivity, and weight management. Furthermore, our findings indicate that vildagliptin influences the reduction of AGE serum levels, which is proposed to be a significant factor contributing to heightened oxidative stress, disruption of insulin signaling, and inflammation. We hypothesize that enhancements in clinical outcomes, including reductions in HbA1c and fasting glucose levels, may be associated with decreased levels of AGEs.

The findings indicate that vildagliptin possesses a favorable profile, characterized by low adverse events and an absence of serious adverse events, positioning it as a viable antihyperglycemic agent that may facilitate glycemic control and address the underlying pathophysiology of insulin resistance.

On the other hand, these interesting findings have several limitations, including a relatively small sample size, a short follow-up period, and a single-center design, which may impact their generalizability and strength.

## Supplementary Information

Below is the link to the electronic supplementary material.

ESM 1 (40.0 KB DOCX)

## Data Availability

Data of the present study are available upon reasonable request from the corresponding author.

## Abbreviations

IR: Insulin resistance
AGE: Advanced glycation end products
AEs: Adverse events
BMI: Body mass index
CVS: Cardiovascular events
DM: Diabetes mellitus
DPP4Is: Dipeptidyl peptidase-4 inhibitors
ERK: Extracellular signal- regulated kinase
FPG: Fasting plasma glucose
HbA1c: Glycated hemoglobin A1c
HOMA-IR: Homeostasis model assessment for insulin resistance
IL-1 and -6: Interleukin-1 and 6
ISRE: Interferon sensitive receptor element
JAK/STAT pathway: Janus kinase and signal transducer and activators of transcription pathway
JNK: Jun N-terminal kinase
MAPK: Mitogen activated protein kinase
NF-kB: Nuclear factor kappa activator beta cells
RAGE: Receptor of advanced glycation end products
RCT: Randomized control trial
ROS: Reactive oxygen species
SAEs: Serious adverse events
SGL2T: Sodium glucose co-transporter 2
T2DM: Type 2 diabetes mellitus
TGB-1: Transforming growth factor B
TNFα: Tumor necrosis factor alpha
VCAM-1: Vascular cell adhesion molecule-1
