# Supplementary material for: Therapeutic impact of vildagliptin vs. gliclazide on insulin resistance and advanced glycated end product levels in newly diagnosed Egyptian diabetics: a randomized controlled trial
Source: Eur J Clin Pharmacol. 2025 Aug 5;81(11):1593–608. doi: 10.1007/s00228-025-03894-8 (PMC12511239; doi:10.1007/s00228-025-03894-8)
Supplement: Supplementary file 1 — (40.0 KB DOCX) [file 228_2025_3894_MOESM1_ESM.docx]

**Supplementary materials**

**Table S1** Effect of adding Vildagliptin or Gliclazide to Metformin on parameter data: Fasting Insulin levels, HbA1c (%), HOMA-IR, BMI values and serum AGEs levels in the two diabetic patients groups after 12 weeks treatment.

| Parameters | **Group I** | **Group II** | **P Value** |
| --- | --- | --- | --- |
| Fasting insulin levels (mIU/ml)  Median (IQR) | 3.4 (3.12 – 3.77) | 3.3 (3 – 3.7) | 0.1610 |
| HbA1c (%)  Median (IQR) | 6.7 (6.22 – 7.07) | 6.3 (6.1 – 6.6)^*^ | < 0.05 |
| HOMA-IR  Median (IQR) | 1.18 (1.08 – 1.29) | 0.77 (0.66 – 0.96)^*^ | < 0.05 |
| BMI (Kg/m^2^)  Median (IQR) | 28.7 (26.8 – 30.8) | 26.4 (24.8 – 29.6)^*^ | < 0.05 |
| Serum AGEs levels (µg/mL)  Median (IQR) | 55.5 (53.25 – 59) | 44 (42.25 – 48)^*^ | < 0.05 |

Group I: Gliclazide 60mg/day + Metformin 1000mg/day, Group II: Vildagliptin 50mg/day + Metformin 1000mg/day. Results were presented as median and interquartile range (IQR) and assessed using Man Whitney U test. BMI: Body mass index, HbA1c: glycated hemoglobin. AGEs: advanced glycated end products. *Significantly distinct from group I at p<0.05.

**Table S2** Effect of adding Vildagliptin or Gliclazide to Metformin on percentage change of body weight in diabetic patients

| Parameter  Treatment | Body weight (Kg) | | |
| --- | --- | --- | --- |
|  | Baseline | Final | Change (%) |
| **Group I** | 80.75 (74.25 - 90) | 83.50 (76.25 - 92) | 2.12 (0 - 3.78) |
| **Group II** | 80 (71.25 - 89) | 77.75 (70 - 85) **^*^** | -2.58 (-4.528 - 0) **^#^** |

Group I: patients received Gliclazide 60mg daily and Metformin 1000 mg daily and Group II; patients received Vildagliptin 50 mg daily and Metformin 1000 mg daily The Mann-Whitney U test was used to examine the data, which was presented as median values with interquartile ranges. Percentage change in body weight was estimated utilizing a formula: % change BWt = [(final BWt - baseline BWt)/baseline BWt] x 100. * significantly distinct from group I at P < 0.05. ^#^ significantly distinct from group I at P < 0.05.
